# Application of a novel lytic phage vB_EcoM_SQ17 for the biocontrol of Enterohemorrhagic *Escherichia coli* O157:H7 and Enterotoxigenic *E. coli* in food matrices

**DOI:** 10.3389/fmicb.2022.929005

**Published:** 2022-08-05

**Authors:** Yan Zhou, Qiyang Wan, Hongduo Bao, Yonghao Guo, Shujiao Zhu, Hui Zhang, Maoda Pang, Ran Wang

**Affiliations:** ^1^School of Food and Biological Engineering, Jiangsu University, Zhenjiang, China; ^2^Jiangsu Key Laboratory for Food Quality and Safety-State Key Laboratory Cultivation Base, Ministry of Science and Technology, Institute of Food Safety and Nutrition, Jiangsu Academy of Agricultural Sciences, Nanjing, China

**Keywords:** phage, EHEC O157:H7, ETEC, biocontrol, foodborne pathogen, foods

## Abstract

Enterohemorrhagic *Escherichia coli* (EHEC) O157:H7 and Enterotoxigenic *E. coli* (ETEC) are important foodborne pathogens, causing serious food poisoning outbreaks worldwide. Bacteriophages, as novel antibacterial agents, have been increasingly exploited to control foodborne pathogens. In this study, a novel broad-host range lytic phage vB_EcoM_SQ17 (SQ17), was isolated, characterized, and evaluated for its potential to control bacterial counts *in vitro* and in three different food matrices (milk, raw beef, and fresh lettuce). Phage SQ17 was capable of infecting EHEC O157:H7, ETEC, and other *E. coli* strains. Morphology, one-step growth, and stability assay showed that phage SQ17 belongs to the *Caudovirales* order, *Myoviridae* family, and *Mosigvirus* genus. It has a short latent period of 10 min, a burst size of 71 PFU/infected cell, high stability between pH 4 to 12 as well as thermostability between 30°C and 60°C for 60 min. Genome sequencing analysis revealed that the genome of SQ17 does not contain any genes associated with antibiotic resistance, toxins, lysogeny, or virulence factors, indicating the potential safe application of phage SQ17 in the food industry. In Luria-Bertani (LB) medium, phage SQ17 significantly decreased the viable counts of EHEC O157:H7 by more than 2.40 log CFU/ml (*p* < 0.05) after 6 h of incubation at 37°C. Phage SQ17 showed great potential to be applied for biocontrol of EHEC O157:H7 in milk and raw beef. In fresh lettuce, treatment with SQ17 also resulted in significant reduction of viable cell counts of EHEC O157:H7 and ETEC at both 4°C and 25°C. Our results demonstrate that SQ17 is a good candidate for application as an EHEC O157:H7 and ETEC biocontrol agent in the processing stages of food production and food preservation.

## Introduction

Foodborne disease is still a serious and underreported public health problem, causing high financial costs to human health and the food industry (Harris et al., 2014; Sites et al., 2017). Food contamination caused by foodborne pathogens is common in developed and developing countries (Senapati et al., 2014). Milk and dairy products are widely consumed by more than 6 billion people worldwide (Vors et al., 2020). Beef is the third most consumed meat in the world, accounting for about 25% of the total meat market (Cui et al., 2017). Furthermore, in China, the consumption of beef and dairy products is increasing much faster than the consumption of pork and poultry meat (Bai et al., 2018). However, milk and beef may be a risk for public health because they can be easily contaminated with various foodborne pathogens. These pathogens can contaminate raw milk during milking or *via* environmental contamination further downstream during processing (Mclean et al., 2013). In addition to milk and beef, lettuce and other fresh vegetables can be exposed to antibiotic-resistant *Escherichia coli* (*E. coli*) and other pathogens through irrigation (Flaherty et al., 2019).

Enterohemorrhagic *E. coli* (EHEC) O157:H7 and Enterotoxigenic *E. coli* (ETEC) are important foodborne pathogens of public health concern that are frequently isolated from agricultural products and can cause food poisoning (Sajed et al., 2016; Zhou et al., 2021b; Dewanggana et al., 2022). EHEC O157:H7, in particular, is one of the major foodborne pathogens worldwide and can cause fatal infections. Even a very low dose (50–100 CFU/g or ml) of EHEC O157:H7 can cause serious human diseases (Forghani et al., 2018; Puligundla and Lim, 2022). EHEC O157:H7 infection is characterized by severe hemorrhagic colitis and hemolytic uremic syndrome (HUS) in humans (Li et al., 2021). Further underscoring the seriousness of this pathogen, EHEC O157:H7 resides in environmental sources (e.g., water and soil) and can contaminate various agricultural food products such as milk, beef, and vegetables. Previous studies showed that foodborne transmission accounted for 85% of the estimated 73,000 cases of *E. coli* O157:H7 infection per year in the United States (Jay et al., 2004). Likewise, ETEC is the most common and important cause of *E. coli* diarrhea in farm animals, infants, and travelers from developed to underdeveloped countries (Okoh and Osode, 2008; Dubreuil and Schifferli, 2016). Foodborne diarrheal disease caused by ETEC infection is primarily acquired *via* contaminated food and water (Okoh and Osode, 2008; Havelaar et al., 2015; Khalil et al., 2016).

Chemical (e.g., antibiotics, disinfectants, and organic acids) and physical (e.g., irradiation, pasteurization, pulsed electric field, and high-pressure processing) control strategies have been widely applied to reduce and inhibit EHEC O157:H7 in the food industry (Chun et al., 2017; Minh et al., 2020; Guo et al., 2021; Puligundla and Lim, 2022). However, these methods suffer from severe major weaknesses (Minh et al., 2020). Use of antibiotics in foods is not permitted, whereas chemical agents may have adverse effects due to remaining chemical residues (Bacanli and Başaran, 2019; Ma et al., 2021). Importantly, antibiotics used for agricultural food products such as beef and milk can transmit to humans through the consumption of these foods (Zhan et al., 2018; Bacanli and Başaran, 2019). Moreover, the ever-increasing emergence of multidrug-resistant bacteria in recent years has resulted in the treatment failure of traditional antibiotics in bacterial infection, leaving few alternatives (Fulle et al., 2015; Son et al., 2018; Gambino and Brøndsted, 2021). Shebs et al. reported that treatment with organic acids is less effective than bacteriophages in decreasing *E. coli* O157: H7 contamination of beef, even under aerobic conditions (Shebs et al., 2020). Further, it has been demonstrated that *E. coli* strains may develop resistance to commonly used organic acids (Toshinobu et al., 1998; Shebs et al., 2020). Physical control strategies, such as irradiation, may destroy the nutrients in foods or require high costs. Therefore, these strategies are not applicable for many kinds of foods (Minh et al., 2020; Puligundla and Lim, 2022). As a result, an alternative approach is urgently needed to control EHEC O157:H7 and ETEC contamination in foods.

Bacteriophages (phages) are bacterial viruses that exist as the most abundant and diverse biological entities on the planet (Zhou et al., 2021a; Chevallereau et al., 2022). Lytic (virulent) phages are viruses that can invade bacterial cells, disrupt the bacterial metabolism, and cause the cell to lyse (Walker et al., 2021). Due to these properties, lytic phages can be used as biocontrol agents for treating bacterial infections in humans and animals, and are recognized as promising alternatives for the decontamination of foodborne pathogens, such as EHEC O157:H7 (Son et al., 2018; Minh et al., 2020). Therefore, phage-based biocontrol strategies are receiving greater interest for their actions against EHEC O157:H7 and ETEC, with the goal to block the source of these contaminations in foods (Sillankorva et al., 2012; Chun et al., 2017). Encouragingly, phage products have been granted Generally Recognized As Safe (GRAS) by the United States Food and Drug Administration (FDA) to apply to human food since 2006 (Chibeu et al., 2013; Guo et al., 2021). Moreover, phage products approved by the FDA have been developed to control *E. coli* O157:H7, including PhageGuard E™, EcoShield™, and Finalyse™ (Carter et al., 2012; Sillankorva et al., 2012; Shebs et al., 2020).

In this study, we isolated and characterized a novel lytic broad-host range EHEC O157:H7 phage vB_EcoM_SQ17 (SQ17) from sewage of a commercial pig farm in the Jiangsu province, China. The newly isolated phage has been submitted to the China Center for Type Culture Collection (CCTCC), with the accession number CCTCC M 20211003. The genome sequence of phage SQ17 suggests the phage belongs to the *Caudovirales* order, *Myoviridae* family, and *Mosigvirus* genus. Phage SQ17 was found to reduce EHEC O157:H7 contamination of milk, raw beef, and fresh lettuce, as well as ETEC contamination of fresh lettuce. Based on the results of host range, lytic activity, stability, replication ability, and inhibition effect in different food matrices, phage SQ17 could be used to control EHEC O157:H7 and ETEC contamination in food production.

## Materials and methods

### Bacterial strains and culture conditions

The bacterial strains used in this study and their characteristics are presented in Table 1. EHEC O157:H7 EO157-1 was used for phage isolation, purification, and propagation, whereas all other strains were used for phage host range determination. EHEC O157:H7 EO157-1 and ETEC EK99-F41 were used for phage application in food matrices. EHEC O157:H7 EO157-1 is resistant to penicillin, vancomycin, and kanamycin. Bacteria were grown aerobically in Luria-Bertani (LB) medium (Oxoid Ltd) at 37°C and stored in 15% glycerol at −80°C until used. For the double agar overlay plaque assay, LB with an additional 0.6% (wt/vol) agar was used to make soft-agar plates.

**Table 1:** Host range analysis of vB_EcoM_SQ17.

Bacterial strains	Toxin genes/serotype	Source	Plaque^e^
EHEC O157:H7			
ATCC43889	*stx2* ^+^	ATCC^a^	+
ATCC35150	*eaeA* ^+^ *stx1* ^+^ *stx2* ^+^	ATCC^a^	+
ATCC43894	*stx1* ^+^ *stx2* ^+^	ATCC^a^	+
ATCC700728	*stx1* ^−^ *stx2* ^−^	ATCC^a^	+
CICC21530	*eaeA* ^+^ *stx1* ^+^ *stx2* ^+^ *hly* ^+^	CICC^b^	+
EO157-1	*stx1* ^−^ *stx2* ^−^	Sewage, this study	+
86–24	*stx1* ^−^ *stx2* ^+^	Laboratory stock^c^	+
363	*stx1* ^−^ *stx2* ^+^	Laboratory stock^c^	+
47	*stx1* ^−^ *stx2* ^+^	Laboratory stock^c^	+
ETEC			
EK99-F41	*k99*^+^*f41*^+^, *stI*^+^	Laboratory stock^c^	+
C83698	O9:K91; K88ac	Swine^d^	+
C83558	O9:K88ac	Swine^d^	+
*Escherichia coli*			
CVCC249	O1	Chicken liver^c^	+
ATCC25922	O1	ATCC^a^	+
BL21		Laboratory stock^c^	+
ATCC35218	*stx1* ^−^ *stx2* ^−^	ATCC^a^	+
*Salmonella typhimurium*			
SalRP04	Typhimurium	Sewage, this study	−
SalRP05	Typhimurium	Sewage, this study	−
*Salmonella enteritidis*			
SalRP17	Enteritidis	Sewage, this study	−
SalRP18	Enteritidis	Sewage, this study	−

a Purchased from the American Type Culture Collection (ATCC).

b Purchased from China Center of Industrial Culture Collection (CICC).

c Zhou et al. (2015).

d Purchased from the China Institute of Veterinary Drugs Control.

e +, formation of phage plaques; −, no formation of phage plaques.

### Phage isolation, purification, and preparation

For phage isolation, several sewage samples were collected from a commercial pig farm in Jiangsu province, China. These samples were used to isolate EHEC O157:H7-specific phages according to the methods described by Zhou et al. with minor modifications (Zhou et al., 2015). Briefly, 5 ml of diluted sewage samples was combined with 5 ml of 2 × LB medium and 500 μl (~8 log CFU/ml) of EHEC O157:H7 EO157-1, and incubated at 37°C for 24 h with constant shaking at 120 rpm. Cultures were then centrifuged at 10,000 × *g* for 10 min, and supernatants were filtered through a 0.22-μm pore size membrane (Merck Millipore Ltd., Ireland) to remove bacterial debris. Ten microliter of the supernatants was used for phage isolation using the spot test. Single plaques of lysis-positive supernatants were isolated and purified using the double agar overlay plaque assay for at least five rounds of amplification (Bao et al., 2019).

Phage was propagated by the phage lysate method, which was performed as previously described (Zhou et al., 2021a). To purify the phage particles, NaCl and PEG 8000 (Amresco, Solon, Ohio, USA) were added to the phage lysate to reach a final concentration of 0.5 M and 10% (w/v), respectively. Finally, the phage particles were precipitated by centrifugation at 11,000 × *g* for 10 min at 4°C and dissolved in SM buffer (5.8 g/L of NaCl, 2.0 g/L of MgSO_4_, 50 ml/L of 1 M Tris, pH 7.5, and 5 ml/L of pre-sterilized 2% gelatin). For food application, phage was prepared by centrifugation of the phage lysate at 11,000 × *g* for 10 min at 4°C and the supernatant was filtered through a 0.22 μm pore size membrane (Merck Millipore Ltd., Ireland), then exchanged to SM buffer by ultrafiltration on a Millipore Amicon Ultra centrifugal filter unit (30 kDa MWCO). The double agar overlay plaque assay was used to determine phage titers. The prepared phage particles were stored at 4°C for further experiments.

### Transmission electron microscopy

Phage samples were deposited onto carbon-coated copper grids and allowed to adsorb for 15 min. The phage particles were negatively stained using 2% (weight/volume) potassium phosphotungstate (pH 7.2) and examined using an H-7650 transmission electron microscope (Hitachi High-Technologies, Tokyo, Japan). Ten virions were examined for the measurement of the phage sizes.

### One-step growth curve

One-step growth curves were performed as previously described (Bao et al., 2019). Briefly, logarithmic growth of strain EO157-1 (7.11 log CFU/ml) was added to phage SQ17 culture at a multiplicity of infection (MOI) of 0.1 and incubated at 37°C for 15 min, followed by centrifugation at 12,000 × *g* for 1 min to remove unabsorbed free phage. The pellet was resuspended in 10 ml LB medium, followed by incubation at 37°C with 180 rpm. Samples (120 μl) were collected as indicated for up to 120 min. Plaque-forming unit (PFU) counts (phage titers) were obtained using the double agar overlay plaque assay. The burst size was calculated as the ratio of the final number of liberated phage particles to the initial number of infected bacterial cells.

### Influence of pH and temperature on phage viability

To determine the tolerance of phage particles to different pH values, peptone water was adjusted to pH values ranging from 2 to 13 with solutions of HCl or NaOH and then filtered through a 0.22-μm filter. Next, 10 μl purified phage suspensions (9 log PFU/ml) were added to the 990 μl peptone water of different pHs and incubated at 37°C for 2 h. Subsequently, the titer of each sample was determined by the double agar overlay plaque assay.

To investigate the thermal tolerance of the phage, 1 ml of purified phage suspensions (9 log PFU/ml) was incubated at a variety of temperatures (i.e., 30°C, 40°C, 50°C, 60°C, 70°C, and 80°C) for 30 and 60 min, respectively. The titer of the remaining viable phage particles was determined using the double agar overlay plaque assay.

### Host range determination

Phage SQ17 was tested against 20 strains by the spot test method (Wu et al., 2021) with some modifications in order to determine its host range. The bacterial strains used in this assay are listed in Table 1. In the first step, 100 μl of potential host bacteria (9 log CFU/ml) was overlaid on LB agar medium and then dried at room temperature for 15 min. Then, a dilution series of the purified phage suspension (10 μl, 8 log PFU/ml) were spotted on plates containing the tested bacterial strain. After overnight incubation at 37°C, the interaction of phage SQ17 with the potential host was determined by the presence of phage plaques at the sites of phage application. The presence of phage plaques was considered evidence of bacterial susceptibility to phage-mediated lysis.

### Phage infection assay

For the phage infection assay, an overnight culture of EHEC O157:H7 EO157-1 was diluted with LB medium to ~6–7 log CFU/ml. Next, phage SQ17 was added at MOIs of 0.01, 0.1, 1.0, and 10, then incubated at 37°C without shaking for 6 h. Cultures without the addition of phage SQ17 were used as controls (MOI = 0). One hundred microliter samples were collected at 3 and 6 h, respectively, and the decrease in viable bacterial cells was tested by plating the aliquots on LB agar plates.

Alternatively, an overnight culture of EHEC O157:H7 EO157-1 was diluted with LB medium to an optical density at 600 nm (OD_600nm_) of ~0.47 (7 log CFU/ml). Then, phage SQ17 was added at various MOIs of 0.01, 0.1, 1.0, and 10, and incubated at 37°C without shaking for 6 h. Cultures without the addition of SQ17 were used as controls (MOI = 0). The bacterial growth was monitored at 30 min intervals by measuring OD_600nm_.

### Phage genome isolation and sequencing

The phage DNA was extracted as described previously (Zhou et al., 2015). The DNA concentration was determined spectrophotometrically by measuring the absorbance at a wavelength of 260 nm. Illumina NovaSeq whole-genome sequencing was carried out by Benagen Tech Solutions Co., Ltd. (Wuhan, China), and the sequencing data were assembled using SPAdes. The quality of the phage genome reads was verified using FastQC.^1^ Finally, a single contig, corresponding to the entire phage genome with an average coverage of 546.01×, was generated. Open reading frames (ORFs) were verified using the NCBI ORF Finder.^2^ The functions of the protein sequences were predicted based on homology with known protein sequences using the protein BLASTp server.^3^ The complete genome sequence of phage SQ17 is deposited at GenBank under accession number MW882907. The genome map was constructed using the CGview Server.^4^ Comparisons of complete genome sequences of other phages were performed with Mauve20150226 (Darling et al., 2010).

### Construction of phylogenetic tree

Phylogenetic analysis based on phage short tail fiber protein amino acid sequences was performed using ClustalW in MEGA 6. To construct the phylogenetic tree, the amino acid sequence of the short tail fiber protein (associated with host range for the *Myoviridae* phage family) of phage SQ17 was compared with the sequences of other reference phages within the *Myoviridae* phage family that were deposited in the NCBI database. The neighbor-joining phylogenetic tree was constructed using the Poisson model, and the robustness of the tree topology was assessed by bootstrap analyses based on 1,000 replicates.

### *In vitro* challenge test

The lytic activity of phage SQ17 was examined *in vitro* as previously described (Minh et al., 2020). Briefly, 5 ml of LB medium was inoculated with 100 μl of bacterial culture of EHEC O157:H7 EO157-1 to obtain the final concentration of ~4 log CFU/ml. The culture was then mixed with phage SQ17 suspensions (a final concentration of 8 log PFU/ml) using a MOI of 10^4^, incubated at 4°C and 25°C, respectively. Aliquots of the culture were taken at 0, 4, 8, 12, 24, 48, 72, 96, 120, 144, and 168 h after infection, and viable cell counts were measured on LB agar plates, followed by incubation overnight at 37°C. Bacterial viable counts were determined after overnight incubation.

### Application of phages treatment in contaminated foods

#### Effect of phages against EHEC O157:H7 in milk

EHEC O157:H7 biocontrol experiments using phage SQ17 were conducted at 4°C (refrigeration temperature) and 25°C (room temperature), as described previously with some modifications (Guo et al., 2021; Li et al., 2021). To avoid the interference of raw milk microbiota, we chose ultra-high temperature (UHT) treated bovine milk as a model to evaluate the inhibitory effect of phage SQ17 on EHEC O157:H7. Ultra-high temperature skim milk and whole milk were purchased from a local supermarket in Nanjing, China. Briefly, an overnight culture of strain EO157-1 was harvested and washed twice with 1 × phosphate-buffered saline (PBS). For experimental groups, 5 ml of UHT skim milk (containing 0% milk fat, Yili Milk, China) or UHT whole milk (6% milk fat, Yili Milk, China) was mixed with strain EO157-1 to a final concentration of ~4–5 log CFU/ml. Then, the phages suspensions at high concentrations (a final concentration of ~8 log PFU/ml) were individually added to 5 ml milk using a MOI of 10^3^–10^4^. Milk with SM buffer (no phages) was used as a control. All mixtures were cultured for 168 h (7 days) at 4°C or 96 h (4 days) at 25°C. Aliquots of the cultures were taken at 0, 4, 8, 12, 24, 48, 72, 96, 120, 144, and 168 h after infection, and viable cell counts were measured on LB agar plates, followed by incubation overnight at 37°C.

#### Effect of phages against EHEC O157:H7 in raw beef

Raw beef was purchased from local supermarkets in Nanjing, China. The outer surface of the raw beef was removed with a sterilized knife, followed by cutting into 2 × 2 cm pieces (about 3 g each). The beef samples were then sterilized using 75% ethanol for 5 min to decontaminate the samples. Before inoculation with bacteria, both sides of all beef samples were exposed to UV light for ~40 min to ensure the killing of any possible microbiota present. Raw beef samples were inoculated with 100 μl of EHEC O157:H7 EO157-1 to reach an approximate viable count of 3.7 log CFU/piece and left at room temperature for 30 min to allow the bacteria to interact with the beef surface. The inoculated samples were administered with 100 μl of the phage suspension to reach a final titer of about 8 log PFU/piece (using a MOI of about 10^4^). The control groups were prepared by inoculating samples with EO157-1, followed by SM buffer administration. Samples were incubated for 120 h (5 days) at 4°C or for 48 h (2 days) at 25°C, respectively. All raw beef pieces were taken after 4, 8, 12, 24, 48, 72, 96, and 120 h of incubation for bacterial enumeration. Viable cell counts were measured on MacConkey agar plates, followed by incubation overnight at 37°C.

#### Effect of phages against EHEC O157:H7 or ETEC in fresh lettuce

Fresh lettuce was purchased from local supermarkets in Nanjing, China. Fresh lettuce was cut into 2 × 2 cm square pieces, rinsed with sterile water for twice and one final rinse with 75% ethanol. Both sides of all lettuce samples were then exposed to UV light for ~30 min to ensure killing of any possible microbiota present. Lettuce samples were inoculated with 50 μl of EHEC O157:H7 EO157-1 or ETEC EK99-F41 to reach an approximate viable count of 4 log CFU/piece and left at room temperature for 30 min to allow the bacteria to interact with the lettuce surface. The inoculated samples were administered with 100 μl of the phage suspension to reach a final titer of about 8 log PFU/piece (using an MOI of 10^4^). The control groups were prepared by inoculating samples with EO157-1 or EK99-F41, followed by SM buffer administration. Samples were incubated for 24 h at 4°C and 25°C, respectively. Lettuce pieces were taken after 2, 4, 8, 12, and 24 h of incubation for bacterial enumeration. Viable cell counts were measured on LB agar plates, followed by incubation overnight at 37°C.

### Statistical analysis

All the experiments were repeated three times. Statistical analyses were performed using GraphPad Prism (version 5.0) statistical analysis software (GraphPad Software, Inc., San Diego, CA, United States). The differences between treatment and control groups were analyzed by unpaired, two-tailed Student’s *t*-test. *p*-values of 0.05 or less were considered significant.

## Results

### Isolation of phage SQ17 and its morphology

**Figure 1:**
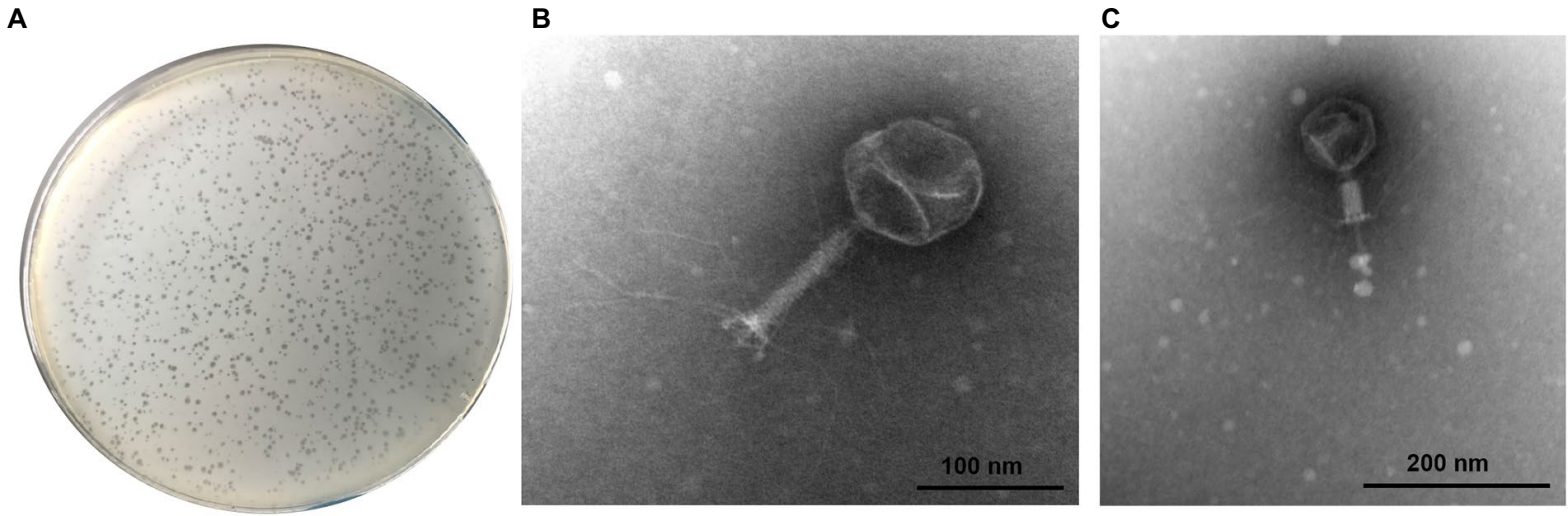
Morphology of EHEC O157:H7 phage SQ17. **(A)** The plaques formed by phage SQ17 on the lawns of EHEC O157:H7 EO157-1. TEM analysis of phage SQ17 with its tail in the non-contracted **(B)** and contracted **(C)** states negatively stained with uranyl acetate (possibly with an empty head). Scale bar, 100 nm.

The isolated phage was capable to produce small and clear plaques (~0.9 mm in diameter) on EHEC O157:H7 EO157-1 culture lawns and was designated as vB_EcoM_SQ17 (SQ17; Figure 1A). Transmission electron microscopy revealed phage SQ17 had a contractile tail and belongs to the *Myoviridae* family (Figures 1B,C). The dimensions of SQ17 were 90.32 ± 2 nm for the head and 16.31 ± 1 nm for the contractile tail (Figures 1B,C).

### One-step growth curve

**Figure 2:**
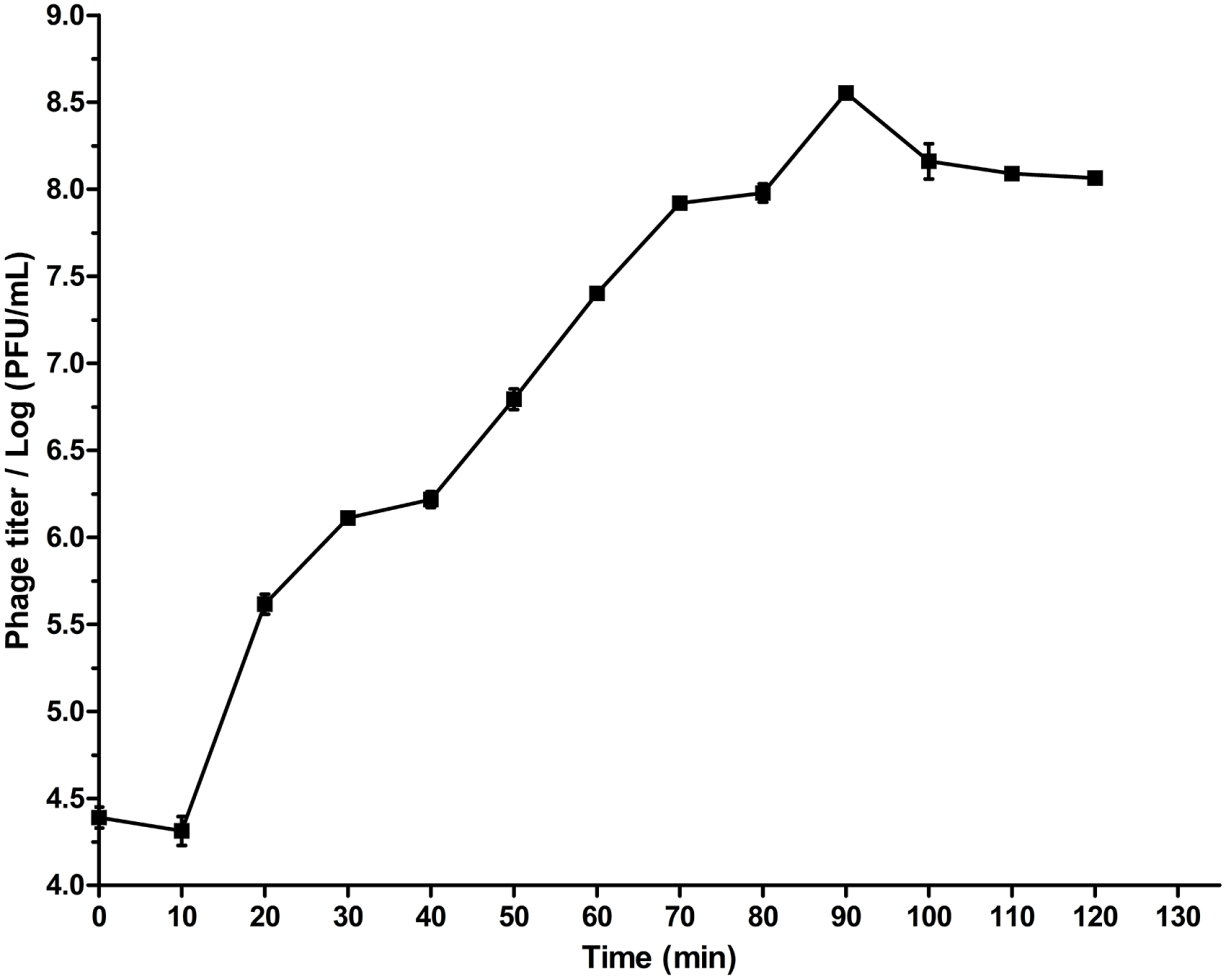
One-step growth curve of phage SQ17 following infection of EHEC O157:H7 EO157-1 at an MOI of 0.1 in LB medium at 37°C. The latent period is 10 min. The values represent the means and standard deviations (SD; *n* = 3).

Analysis of the one-step growth curve showed that the latent period of phage SQ17 was 10 min, after which the number of phage particles rapidly increased. The average burst size was 71 PFU/infected cell (Figure 2).

### pH and thermal stability

**Figure 3:**
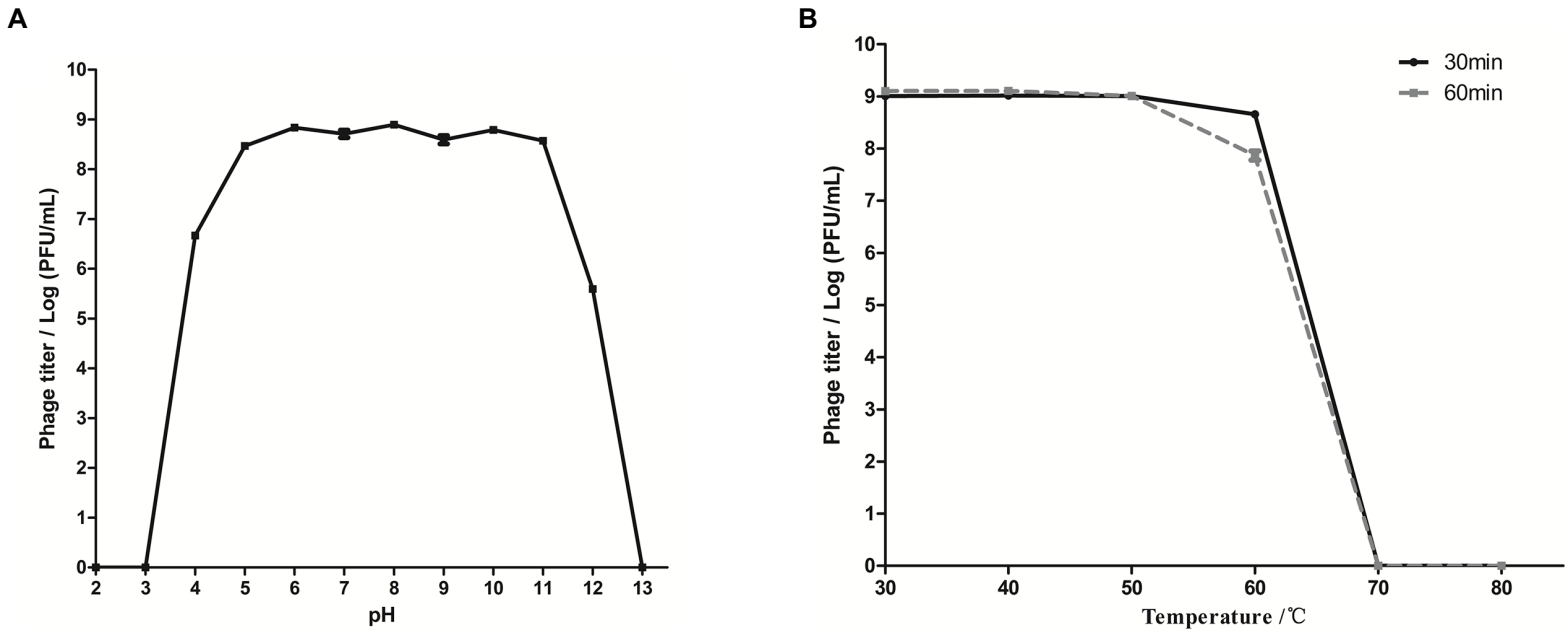
Biological characteristics of phage SQ17 at different pH values and temperatures. Tolerance of phage SQ17 at different pH values **(A)** and temperatures **(B)**. The values represent the means and standard deviations (SD; *n* = 3).

The stability of phage SQ17 exposed to different pHs and temperatures was tested. SQ17 was relatively stable between pH 4–12 (stability rate > 60%), and the stability was >85% at pH 5–11 (Figure 3A). SQ17 was stable from 30°C to 60°C with the stability rate of more than 86% at the elevated temperatures for 60 min, including almost no appreciable loss of infectivity at 60°C. However, no viable phages were detected upon heating at temperatures ≥70°C (Figure 3B). Nevertheless, the result suggests that phage SQ17 was stable and retained infectivity when stored at ambient temperatures.

### Host range

Sixteen *E. coli* strains from various sources were tested to determine the host range of phage SQ17 (Table 1). The spot test assay indicated that SQ17 had a lytic effect on all 16 *E. coli* strains, including EHEC O157:H7 strains, ETEC strains, and other *E. coli* strains (Table 1). However, SQ17 did not infect four representative *Salmonella* spp. strains.

### Lytic activity of phage SQ17 *in vitro*

**Figure 4:**
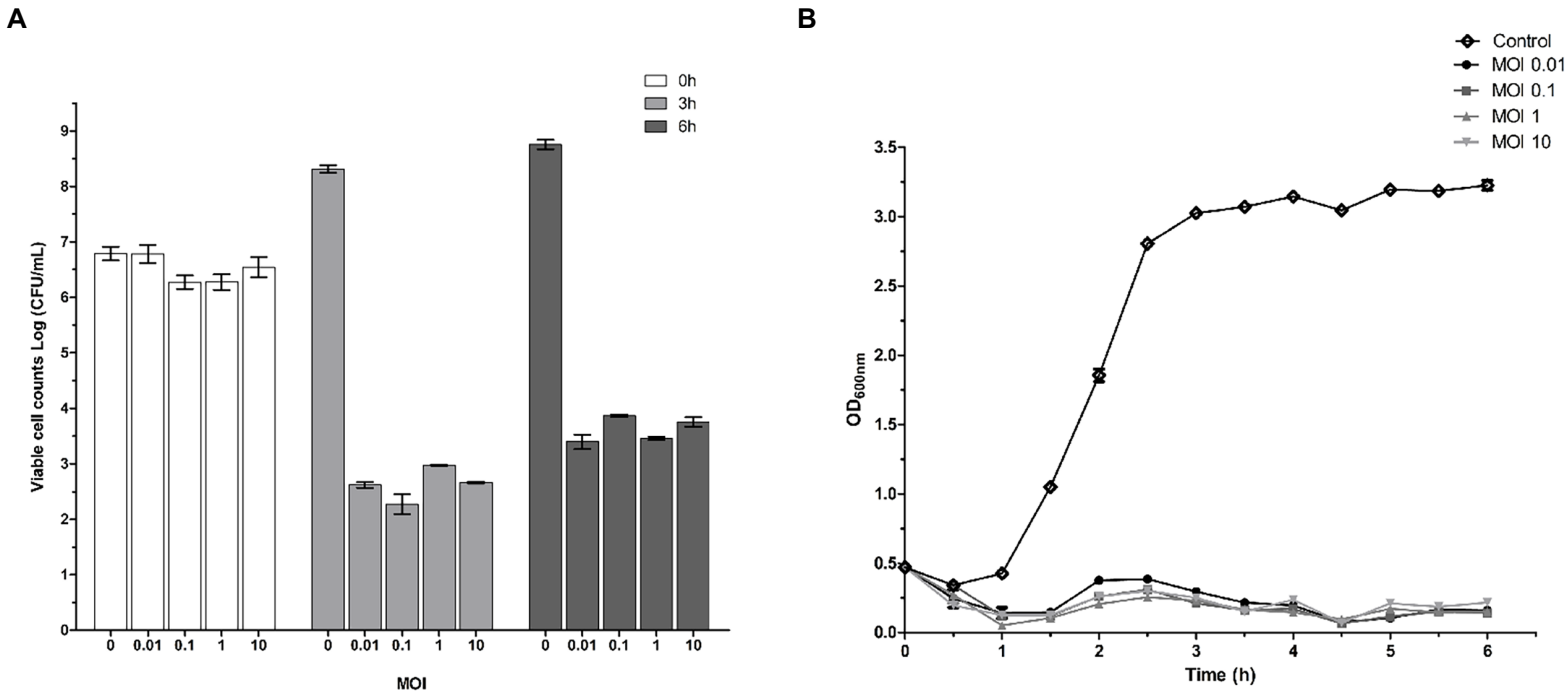
Effect of phage SQ17 on EHEC O157:H7. **(A)** Phage inhibition of viable EHEC O157:H7 EO157-1 cell counts at 0, 3, and 6 h in LB medium at an MOI of 10, 1, 0.1, or 0.01, respectively. **(B)** Lytic activity of phage SQ17 against EHEC O157:H7 EO157-1 in LB medium at an MOI of 10, 1, 0.1, or 0.01. Absorbance measurements at 600 nm were made every 30 min. All cultures were incubated at 37°C. The control contained EHEC O157:H7 EO157-1 only. The values represent the means and standard deviations (SD; *n* = 3).

Phage SQ17 showed significant killing activity at four different MOIs of infection (0.01, 0.1, 1, and 10; *p* < 0.05). Figure 4 shows the effect of phage SQ17 on the viability of EHEC O157:H7 EO157-1 cultured in LB medium at 37°C. In the phage-infected cultures, viable counts of EHEC O157:H7 EO157-1 decreased by 4.16, 4.00, 3.29, and 3.87 log CFU/ml after 3 h of incubation (Figure 4A). Even after 6 h of incubation, the cell concentrations in the treated cultures were 3.39, 2.40, 2.81, and 2.78 log CFU/ml which were lower than the initial cell concentrations (Figure 4A).

A turbidity assay was also used to determine the lytic activity of phage SQ17 against EHEC O157:H7. In the control, EHEC O157:H7 EO157-1 began growing after 1 h of incubation and reached 3.0 OD_600nm_ after 3 h of incubation, then entered the stationary phase. However, bacterial growth was strongly inhibited when infected with phage SQ17 at four different MOIs, 0.01, 0.1, 1, and 10 (*p* < 0.0001; Figure 4B).

### Genome and phylogenetic analysis of phage SQ17

**Figure 5:**
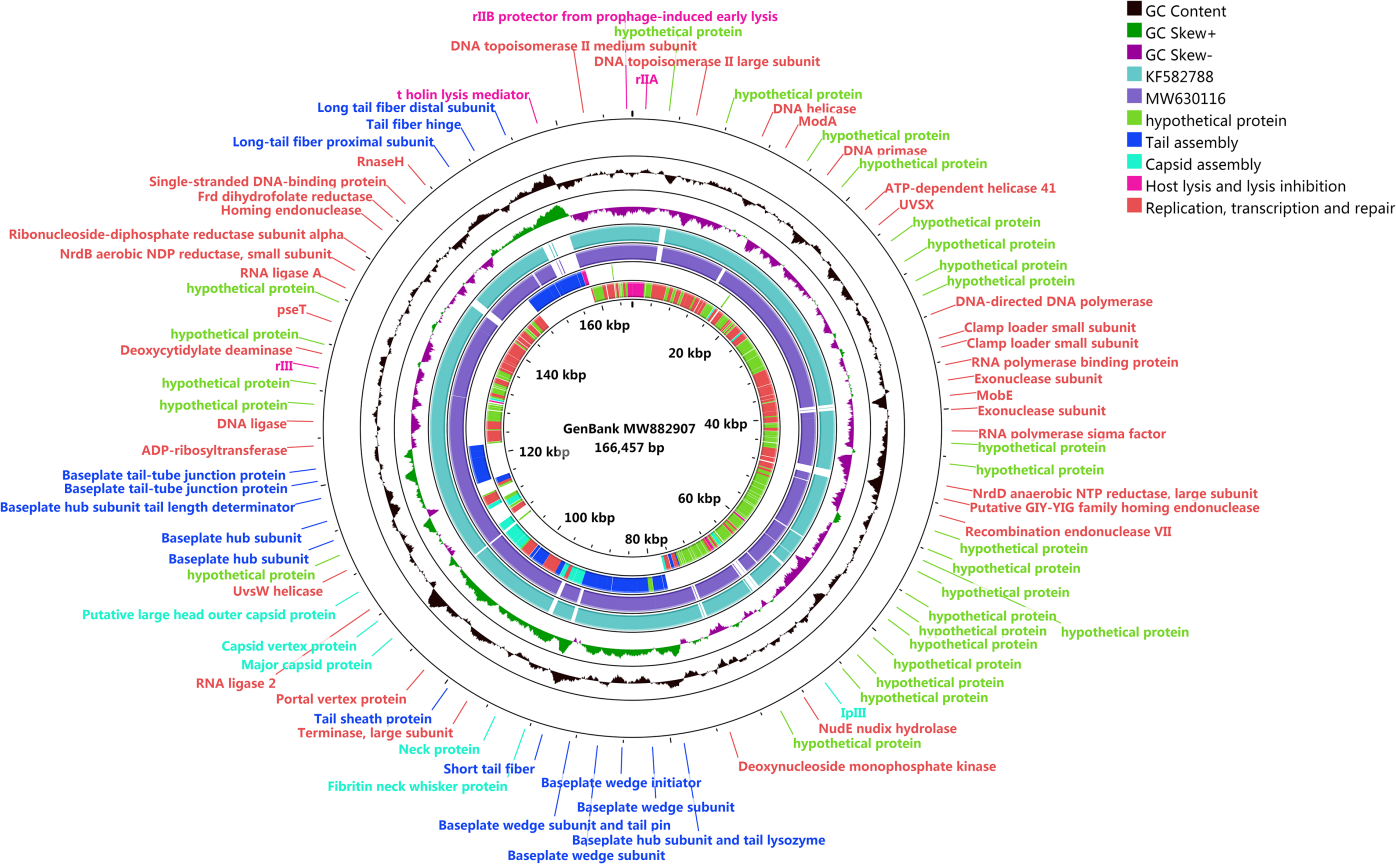
Genome map of phage SQ17. The genome was generated using CGview server beta and is annotated and colored based on predicted molecular functions of identified genes. The center of the genome map provides % GC content (black) and the GCskew+ and skew-are shown in light green and purple, respectively.

The genome of phage SQ17 was found to be linear double-stranded DNA of 166,457 bp with 37.52% GC content. The genome contained 258 putative open reading frames (ORFs). Among them, 136 ORFs (52.7%) were classified as hypothetical proteins, whereas the putative functions of 122 ORFs were predicted (47.3%) based on gene prediction and genome annotation (Supplementary Table S1). These proteins clustered into four functional groups related to tail assembly, capsid assembly, host lysis and lysis inhibition, replication, transcription, and repair (Figure 5). No lysogeny, toxin, antibiotic resistance, or virulence genes were detected by separate searches with lysogenic genes, the Comprehensive Antibiotic Resistance Gene Database^5^ and the Virulence Factor Database.^6^ The genome sequence of phage SQ17 is available in the GenBank database under the accession number (MW882907). A blast search revealed that the genome of phage SQ17 had 97.85%, 97.71%, and 97.2% homology to genomes of *Escherichia* phage vB_EcoM-ZQ3 (query cover 91%; GenBank accession number MW630116), *Escherichia* phage vB_EcoM_JS09 (query cover 92%; GenBank accession number KF582788), and *Escherichia* phage SF (query cover 94%; GenBank accession number NC_055749.1), respectively. Phage SQ17 belongs to the *Caudovirales* order, *Myoviridae* family, and *Mosigvirus* genus based on the phage biological characterization and the genomic information.

**Figure 6:**
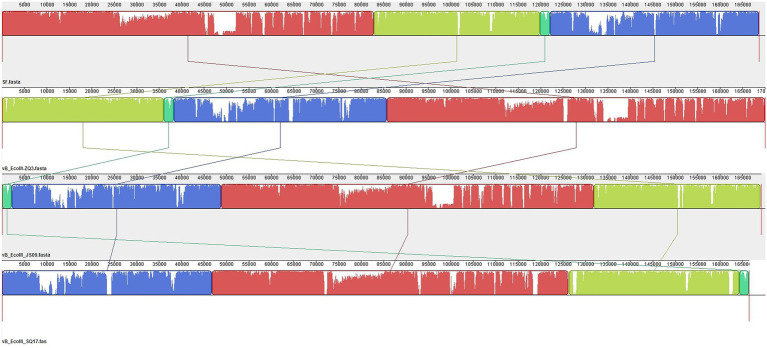
Comparative genomic analysis of phage SQ17 with phages SF, vB_EcoM-ZQ3 and vB_EcoM_JS09 using Mauve. Nucleotide sequence similarity is indicated by the height of the colored bars, while regions that are dissimilar are in white.

**Figure 7:**
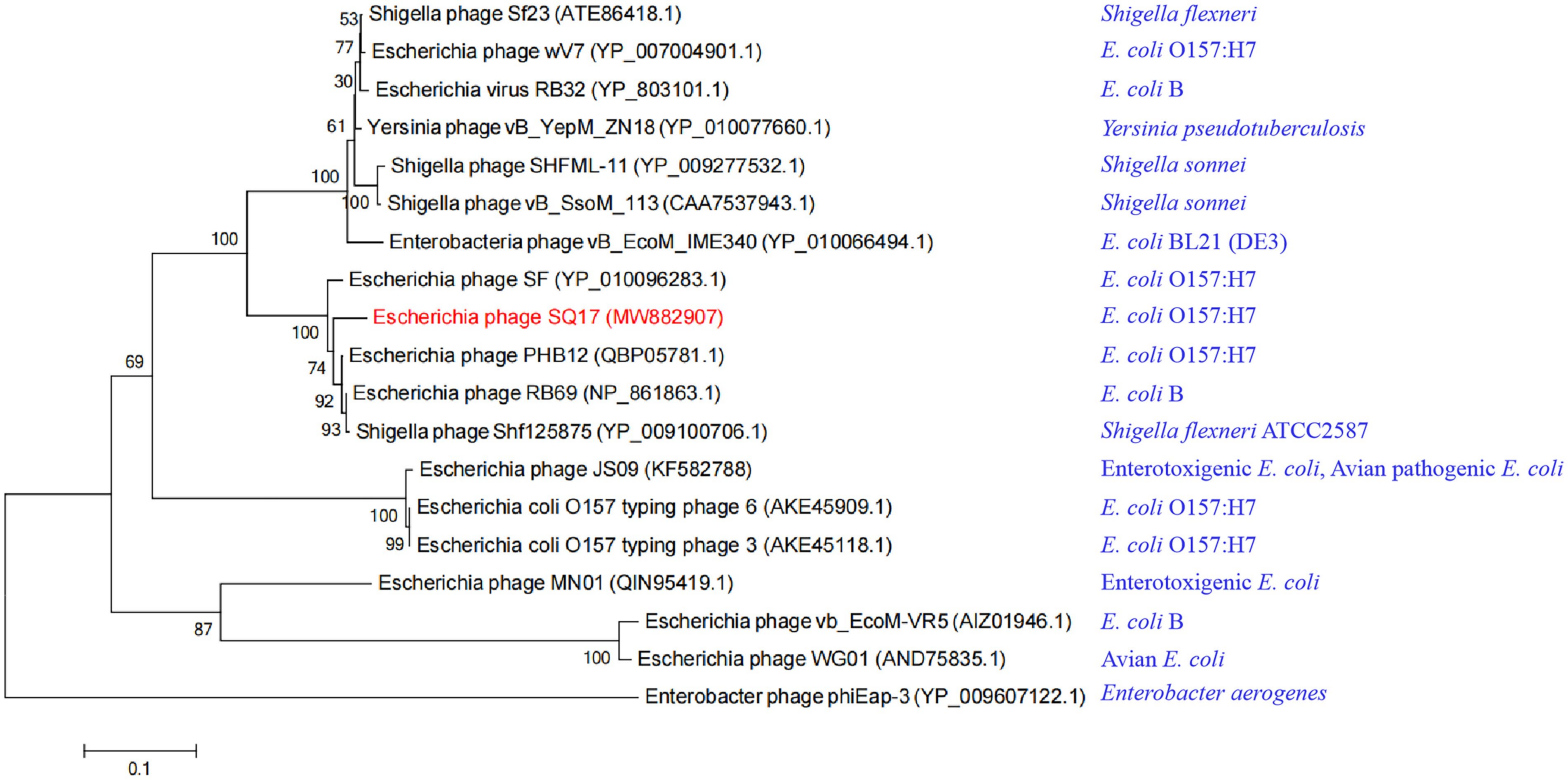
Phylogenetic tree of phages with similar short tail fiber protein sequences to phage SQ17. Host bacteria of phages are in blue.

When we further compared the whole genome sequence of SQ17 with three closely related phages, SF, vB_EcoM-ZQ3, and vB_EcoM_JS09, by mauve analysis, the results showed that all phages had similar gene module arrangements (Figure 6). Moreover, phylogenetic analysis revealed that phage SQ17 clustered in the same phylogenetic sub-branch with other *E. coli* O157:H7 phages when comparing only their short tail fiber proteins (Figure 7). The short tail fiber protein of phage SQ17 was found to be most closely related to those of phages SF and PHB12, which also infect *E. coli* O157:H7.

### Inhibition effect of phage SQ17 on EHEC O157:H7 in LB medium

**Figure 8:**
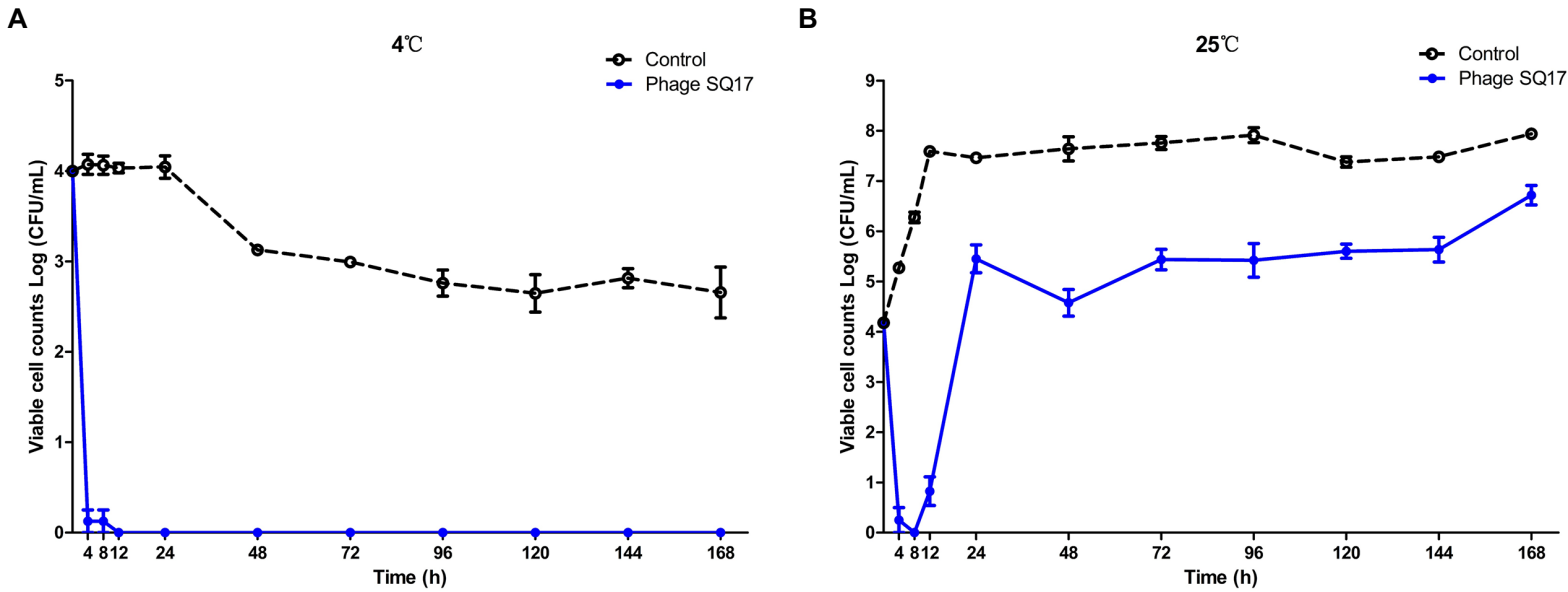
Effect of phage SQ17 against EHEC O157:H7 EO157-1 in LB medium at 4°C **(A)** and 25°C **(B)**. The error bars show the standard error of the mean.

In the presence of phage SQ17, the viable counts of bacterial hosts in the LB medium were significantly reduced (*p* < 0.0001) compared with those of phage-free controls at both 4°C and 25°C (Figure 8). After 4 h of phage treatment, viable counts decreased ~4 log (*p* < 0.05) at 4°C (Figure 8) and the bacterial regrowth was not observed throughout incubation period. However, at 25°C, the viable count increased after 8 h of incubation.

### Inhibition effect of phage SQ17 on EHEC O157:H7 in milk

**Figure 9:**
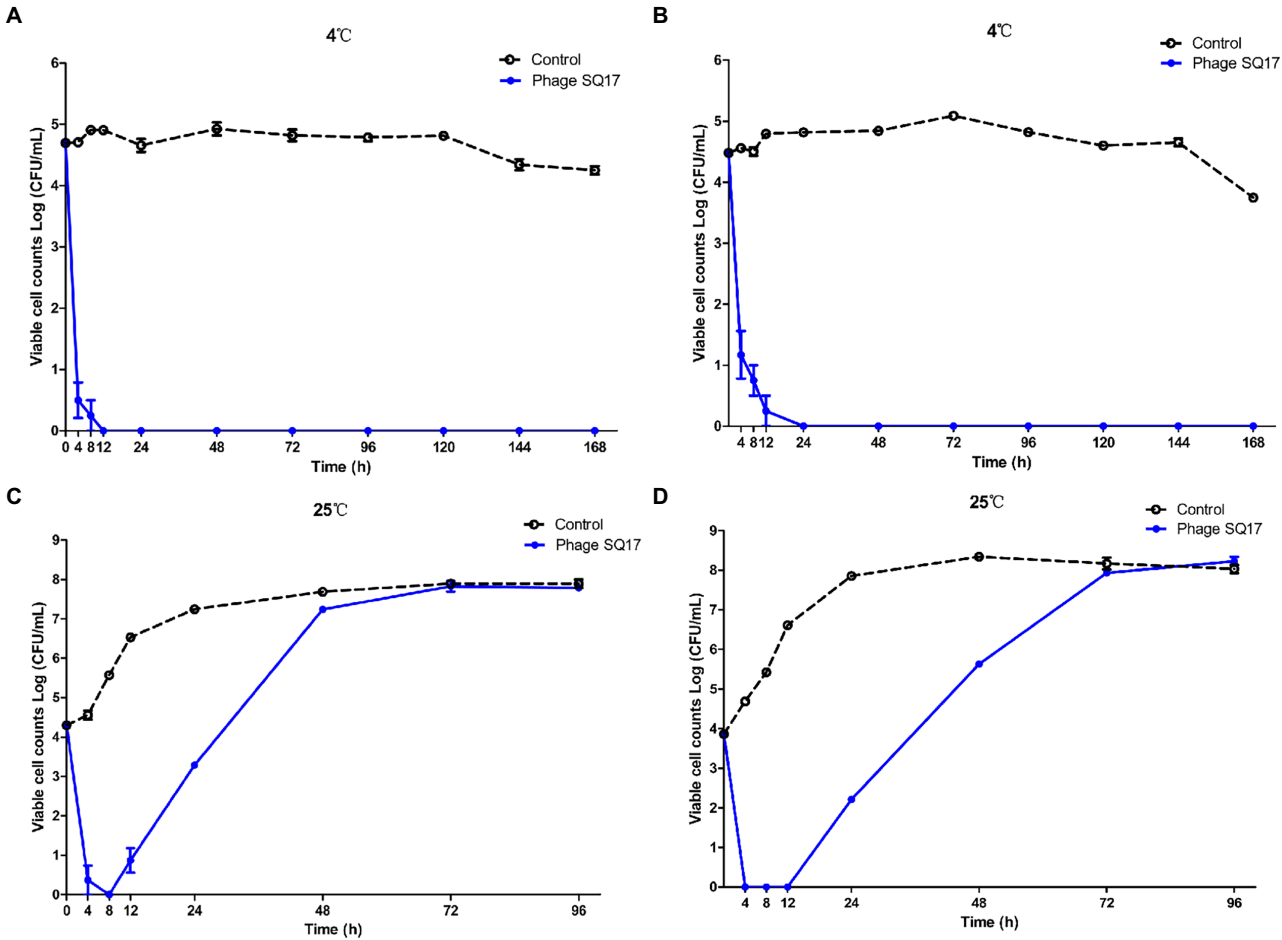
Effect of phage SQ17 against EHEC O157:H7 EO157-1 in milk. The viability of EO157-1 cell in UHT skim milk **(A)** and UHT whole milk **(B)** incubated at 4°C, and in UHT skim milk **(C)** and UHT whole milk **(D)** incubated at 25°C. The error bars show the standard error of the mean.

Phage SQ17 was evaluated for its lytic activity against EHEC O157:H7 in UHT milk at 4°C or 25°C by monitoring the viable bacterial cell counts for 7 or 4 days, respectively. The level of contamination of EHEC O157:H7 strain EO157-1 assayed in skim or whole UHT milk was below the detection limit (<10 CFU/ml) when phage SQ17 was applied at 4°C (Figures 9A,B, respectively). Specifically, viable cell counts of EO157-1 dropped from ~4.7 log CFU/mL to below the detection limit (*p* < 0.0001) after 4 h of exposure in UHT skim milk, and cells were undetectable after 12 h of exposure to phages (Figure 9A). Likewise, viable cell counts of EO157-1 dropped from ~4.5 log CFU/ml to below the detection limit (*p* < 0.0001) after 8 h of exposure in UHT whole milk, and cells were undetectable after 24 h of exposure to phages (Figure 9B). When measurements were extended to 7 days, no viable counts were recorded indicating application of phages resulted in complete sterility (Figures 9A,B).

At a higher temperature of 25°C, viable cell counts of EO157-1 dropped from ~4 log CFU/ml to below the detection limit (*p* < 0.05) after 4 h of exposure in skim and whole UHT milk (Figures 9C,D, respectively). Although bacterial regrowth was observed, after 12 and 24 h of incubation, reductions achieved were ≥3.43 and 1.01 log CFU/ml, respectively (Figures 9C,D). However, after 72 h of incubation, bacterial growth was the same as that of the control group.

### Inhibition effect of phage SQ17 on EHEC O157:H7 in raw beef

**Figure 10:**
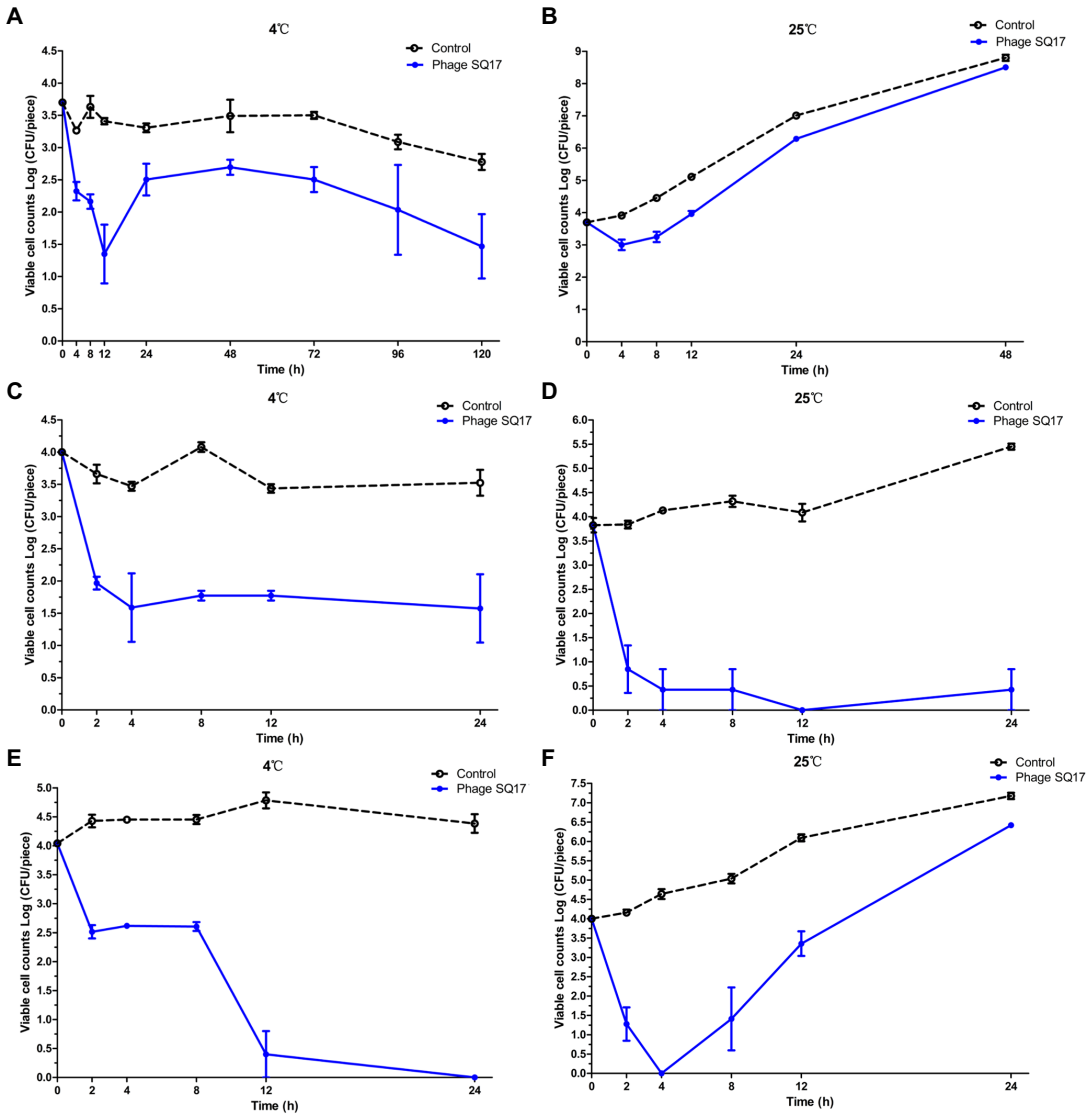
Effect of phage SQ17 against EHEC O157:H7 EO157-1 and ETEC EK99-F41 in solid food matrices. The viability of EO157-1 cell on raw beef incubated at **(A)** 4°C and **(B)** 25°C and on fresh lettuce incubated at **(C)** 4°C and **(D)** 25°C. The viability of EK99-F41 cell on fresh lettuce incubated at **(E)** 4°C and **(F)** 25°C. The error bars show the standard error of the mean.

To determine the bactericidal ability of phage SQ17 in solid foods, raw beef was artificially contaminated with EHEC O157:H7 strain EO157-1 to a final viable cell count of 3.7 log CFU/piece. Phage SQ17 application on raw beef significantly reduced the viable cell counts of EO157-1 by 2.35 log CFU/piece (*p* < 0.05) after 12 h of incubation at 4°C and 0.70 log CFU/piece (*p* < 0.05) after 4 h of incubation at 25°C (Figures 10A,B, respectively). Even after 5 days, viable cell counts of EO157-1 were decreased by 2.23 log CFU/piece at 4°C compared to the control (Figure 10A). However, after 48 h of incubation, the cell concentrations of the phage-treated group were close to that of the control group at 25°C (Figure 10B).

### Inhibition effect of phage SQ17 on EHEC O157:H7 or ETEC in fresh lettuce

Fresh lettuce was artificially contaminated with EHEC O157:H7 strain EO157-1 or ETEC strain EK99-F41 to final viable cell counts of ~4 log CFU/piece. Phage SQ17 application on fresh lettuce significantly reduced the viable cell counts of EO157-1 by 2.23 log CFU/piece (*p* < 0.05) and 3.83 log CFU/piece (*p* < 0.05) after 12 h incubation at 4°C and 25°C, respectively (Figures 10C,D). After 24 h, viable cell counts of EO157-1 decreased by 2.42 log CFU/piece (*p* < 0.05) and 3.40 log CFU/piece (*p* < 0.05) at 4°C and 25°C, respectively (Figures 10C,D). At both 4°C and 25°C, bacterial regrowth was not observed.

In the phage-treated ETEC experiment, phage SQ17 application on fresh lettuce significantly reduced the viable cell counts of EK99-F41 by 3.64 log CFU/piece (*p* < 0.05) after 12 h of incubation at 4°C and 4.00 log CFU/piece (*p* < 0.05) after 4 h of incubation at 25°C (Figures 10E,F). Even after 24 h, viable cell counts of EK99-F41 decreased by 4.04 log CFU/piece (*p* < 0.05) at 4°C (Figure 10E). Between 12 and 24 h, the viable cell counts were almost undetectable at 4°C (Figure 10E). However, at 25°C, the bacterial cell counts began to grow again after 4 h of incubation (Figure 10F).

## Discussion

An important aspect in developing phage-based biocontrol strategies for food application is that the phages used must be safe and strictly lytic (Cong et al., 2021). In this study, a thorough genome analysis was undertaken for phage SQ17 and no evidence of antibiotic resistance, toxins, lysogeny, or virulence factor-related genes was found, suggested that this phage is an excellent candidate for potential food application.

In phage T4, the short tail fiber protein irreversibly binds to the receptor on the host cell surface (Nobrega et al., 2018). According to genome sequence analysis based on the NCBI database, the short tail fiber protein of phage SQ17 has two conserved domains, a phage short tail fiber gp12 domain and a lipopolysaccharide (LPS) receptor-binding domain. Due to the broad host range of phage SQ17, we constructed a phylogenetic tree using the short tail fiber proteins of *E. coli* O157:H7, ETEC, and other *E. coli* phages. Consistent with the results of phylogenetic analysis, BLASTp search results showed the short tail fiber protein of phage SQ17 shares high amino acid sequence identity (>94%) with that of *E. coli* O157:H7 phages (PHB12, SF, and phiE142) and *E. coli* phage (RB69), which may be a key characteristic of SQ17 due to its ability to infect EHEC O157:H7, ETEC, and other *E. coli* strains. Additionally, a comparative analysis of the genomes of phages SQ17, vB_EcoM-ZQ3, vB_EcoM_JS09, and SF revealed that genomic rearrangements could be due to recombination between different phages. This result is in accordance with the remarkable mosaic architecture and the high degree of similarity among phage genomes, likely resulting from their high degree of horizontal gene transfer (Cong et al., 2021; Chevallereau et al., 2022).

Phages normally infect certain bacteria with high specificity (Mo et al., 2013; Switches et al., 2021). From the aspect of a phage-based application, the narrow host range of phages is a major limitation when applied to the food industry (Globus et al., 2017; Minh et al., 2020). To solve this problem, a designed phage cocktail approach has been explored in milk, meat, fruits, and vegetables (Tomat et al., 2018; Petsong et al., 2019; Phothaworn et al., 2020). The major advantage of a phage cocktail is that it can effectively prevent the emergence of phage-resistant mutants (Son et al., 2018). However, in a phage cocktail, the combination of several phages may lead to the recombination and antagonism between phage members, not to mention the increased cost of preparation of the cocktail (Son et al., 2018; Dion et al., 2020; Minh et al., 2020). Therefore, as a complementary method, phages with a broad host range are also under development for phage-based biocontrol of foodborne pathogens (Hagens and Loessner, 2010; Zhang et al., 2021). Along these lines, studies of phages for food application to effectively reduce EHEC O157:H7 contamination have been reported, including phages BECP10, PE37, JN01, and HY01 (Heyn et al., 2016; Son et al., 2018; Li et al., 2021; Park and Park, 2021).

In this study, phage SQ17 was found to possess a broad host range and was found to be capable of infecting EHEC O157:H7, ETEC, and other *E. coli* strains. As one of the major foodborne pathogens, ETEC is also an important cause of bacterial diarrheal illness, mainly infecting travelers and children, especially in developing countries (Madhavan et al., 2014; Kumar et al., 2019; Wang et al., 2019). It can contaminate fresh vegetables and adhere firmly to lettuce through its flagella (Shaw et al., 2011; Luna-Guevara et al., 2019). Thus, both EHEC O157:H7 and ETEC are potential infection hazards in fresh vegetables, particularly when making ready-to-eat salad (Castro-rosas et al., 2012; Luna-Guevara et al., 2019). We found that phage SQ17 was able to reduce EHEC O157:H7 and ETEC contamination in fresh lettuce. This is the first study on a single phage capable of controlling EHEC O157:H7 and ETEC contamination in fresh vegetables.

For food industry applications, phages with a broad host range, short latent period, and large burst size could be desirable candidates (Minh et al., 2020; Luo et al., 2021). Phage SQ17 has a large burst size of 71 PFU/infected cell and, a shorter latent period of 10 min compared with phages HY01, JN01, PS5, and PE37 (Heyn et al., 2016; Son et al., 2018; Minh et al., 2020; Li et al., 2021). These data suggest that SQ17 has a high infection ability and fast replication rate. In fact, phages having prolonged persistence in variable environmental conditions are preferred for application in the food industry (Luo et al., 2021). In this study, we evaluated the performance of phage SQ17 to retain infectivity after incubation at different pHs and temperatures. Generally, temperature and pH are considered significant main factors to evaluate a phage for food application (Jończyk et al., 2011; Minh et al., 2020). Phage SQ17 showed high stability from pH 5 to 11 and at a wide temperature stability range from 30°C to 60°C for 60 min. Even at pHs 4 and 12, phage SQ17 still retained almost 65% activity. Compared with *E. coli* O157:H7 phages reported previously, phage SQ17 showed almost the same tolerance to harsh environments with a slight difference (Lee et al., 2020; Minh et al., 2020; Li et al., 2021; Park and Park, 2021). Our results indicated that phage SQ17 may have a favorable shelf life and good stability when applied in a food storage environment.

Milk, beef, and lettuce are the main sources of exposure and ingestion of EHEC O157:H7 by humans (Lu and Breidt, 2015; Lee et al., 2020; Li et al., 2021; Park and Park, 2021). Therefore, we determined the potential use and efficacy of SQ17 in controlling EHEC O157:H7 in these foods. Our results showed that phage SQ17 has a strong bactericidal ability in milk, and on raw beef and fresh lettuce. These foods were artificially inoculated with EHEC O157:H7, followed by phage treatment at 4°C and 25°C to simulate refrigeration and room temperatures. After 12 h of phage infection, viable counts of pre-inoculated EHEC O157:H7 were significantly reduced for all three tested food matrices. The viable count reduction we observed varied between 4.7 log CFU/mL for EHEC O157:H7 in milk, about 2.35 log CFU/piece in beef, and 3.83 log CFU/piece in lettuce. Our findings are consistent with previous studies (Guo et al., 2021), which found higher efficacy for phage in milk (liquid food) than on beef or lettuce (solid foods) at 4°C. The differences may be attributable to better movement and attachment of phage to bacteria in a liquid environment opposed to a solid food matrix. Our results also showed that milk fat has little influence on phage SQ17 treatment. In UHT whole milk and UHT skim milk, the variation curves of bacterial counts were almost the same when treated with phage SQ17 at 4°C and 25°C. These results are similar to phage PS5, which was shown to reduce the viable counts of *E. coli* O157:H7_Cyan to under the detection limit (<10 CFU/ml) after 2 h at both 4°C and 24°C in whole fat pasteurized milk (Minh et al., 2020). In a related study on lettuce contamination, phage DW-EC only reduced 46.88% of ETEC on lettuce after 1 day of incubation at 4°C (Dewanggana et al., 2022). However, in our findings, no ETEC was detected in lettuce after 1 day of incubation with phage SQ17 at 4°C. Moreover, no one has reported that a single phage is able to infect both EHEC O157:H7 and ETEC on fresh lettuce.

The application of phage SQ17 was particularly effective in reducing contamination of milk at refrigeration temperature, as it decreased the number of EHEC O157:H7 below the detection limit, which may be attributed to the high stability of the phages and the reduced growth rate of their bacterial host at 4°C. It is reported that the efficacy of phage-based biocontrol strongly depends on temperature (Minh et al., 2020). At refrigeration temperatures, the growth of bacteria is slowed; hence, the effect of phage treatment would be affected by the low temperature (Minh et al., 2020). Similar to the previous study on food application (Minh et al., 2020), we also found bacterial regrowth at room temperature. Higher temperature (25°C) is one of the more favorable conditions for the growth of *E. coli*, which consequently leads to less efficacy of phage SQ17. Nonetheless, it is not recommended to store or process fresh foods at high temperatures for long periods of time in the food industry. Next, we will focus on investigating the synergistic antibacterial effect of phage and natural bactericidal agents to prevent the appearance of bacteria refractory to phage treatment.

## Data availability statement

The datasets presented in this study can be found in online repositories. The names of the repository/repositories and accession number(s) can be found in the article/Supplementary material.

## Author contributions

YZ: conceptualization, funding acquisition, data curation, investigation, resources, software, supervision, validation, visualization, and writing–original draft. QW: data curation, formal analysis, investigation, methodology, software, validation, and visualization. HB: data curation, formal analysis, investigation, methodology, project administration, resources, and writing–review and editing. YG: data curation and methodology. SZ: data curation and methodology. HZ: methodology. MP: software. RW: supervision. All authors contributed to the article and approved the submitted version.

## Funding

This work was supported by the National Natural Science Foundation of China (no. 31972764 to YZ).

## Conflict of interest

The authors declare that the research was conducted in the absence of any commercial or financial relationships that could be construed as a potential conflict of interest.

## Publisher’s note

All claims expressed in this article are solely those of the authors and do not necessarily represent those of their affiliated organizations, or those of the publisher, the editors and the reviewers. Any product that may be evaluated in this article, or claim that may be made by its manufacturer, is not guaranteed or endorsed by the publisher.

## Footnotes

^1^ https://www.bioinformatics.babraham.ac.uk/projects/fastqc/

^2^ https://www.ncbi.nlm.nih.gov/orffinder/

^3^ https://blast.ncbi.nlm.nih.gov/Blast.cgi

^4^ http://cgview.ca/

^5^ https://card.mcmaster.ca/

^6^ http://www.mgc.ac.cn/VFs/main.htm

## Supplementary material

The Supplementary material for this article can be found online at: https://www.frontiersin.org/articles/10.3389/fmicb. 2022.929005/full#supplementary-material
