# Supplementary material for: Application of a novel lytic phage vB_EcoM_SQ17 for the biocontrol of Enterohemorrhagic Escherichia coli O157:H7 and Enterotoxigenic E. coli in food matrices
Source: Front Microbiol. 2022 Aug 5;13:929005. doi: 10.3389/fmicb.2022.929005 (PMC9389114; doi:10.3389/fmicb.2022.929005)
Supplement: Supplementary file 1 [file Table_1.doc]

**Application of a novel lytic phage vB_EcoM_SQ17 for the biocontrol of Enterohemorrhagic *Escherichia coli* O157:H7 and Enterotoxigenic *Escherichia coli* in food matrices**

Yan Zhou^1,2^*, Qiyang Wan^1,2^, Hongduo Bao^2^, Yonghao Guo^2^, Shujiao Zhu^2^, Hui Zhang^2^, Maoda Pang^2^, Ran Wang^2^*

^1^ School of Food and Biological Engineering, Jiangsu University, Zhenjiang, China

^2^ Jiangsu Key Laboratory for Food Quality and Safety-State Key Laboratory Cultivation Base, Ministry of Science and Technology, Institute of Food Safety and Nutrition, Jiangsu Academy of Agricultural Sciences, Nanjing, China

*Corresponding author Yan Zhou: E-mail: zhouyan2007@jaas.ac.cn

or Ran Wang: [ranwang@jaas.ac.cn](mailto:ranwang@jaas.ac.cn)

**Supplementary data**

**Table S1**

Genomic annotation of functional ORFs of bacteriophage SQ17.

| ORF | Start | End | Strand | | Function |
| --- | --- | --- | --- | --- | --- |
|  | 1 | 2214 | - | rIIA protector from prophage-induced early lysis | |
|  | 2227 | 2427 | - | Hypothetical protein | |
|  | 2520 | 3620 | - | Hypothetical protein | |
|  | 3663 | 4460 | - | Putative HNH homing endonuclease | |
|  | 4460 | 6280 | - | DNA topoisomerase II large subunit | |
|  | 6344 | 6769 | - | Hypothetical protein | |
|  | 6772 | 6951 | - | Hypothetical protein | |
|  | 6954 | 7364 | - | mRNA metabolism modulator | |
|  | 7364 | 7591 | - | Modifier of suppressor tRNAs | |
|  | 7649 | 8173 | - | Hypothetical protein | |
|  | 8233 | 8652 | - | Modifier of transcription | |
|  | 8662 | 9117 | - | Modifier of transcription | |
|  | 9179 | 9673 | - | Hypothetical protein | |
|  | 9740 | 10417 | - | Exonuclease | |
|  | 10427 | 11740 | - | DNA helicase | |
|  | 11737 | 12048 | - | Hypothetical protein | |
|  | 12051 | 12797 | - | Putative anti-sigma factor | |
|  | 12950 | 13558 | - | ModA RNA polymerase ADP-ribosylase | |
|  | 13616 | 14197 | - | ModB RNA polymerase ADP-ribosylase | |
|  | 14253 | 14417 | - | Hypothetical protein | |
|  | 14414 | 14593 | - | Hypothetical protein | |
|  | 14595 | 15059 | - | Hypothetical protein | |
|  | 15059 | 15238 | - | Hypothetical protein | |
|  | 15235 | 15462 | - | Hypothetical protein | |
|  | 15449 | 15676 | - | Hypothetical protein | |
|  | 15781 | 16017 | - | Small outer capsid protein | |
|  | 16063 | 16584 | - | dCTP pyrophosphatase | |
|  | 16674 | 16871 | + | Hypothetical protein | |
|  | 16868 | 17890 | - | DNA primase | |
|  | 17930 | 18397 | - | Hypothetical protein | |
|  | 18417 | 18614 | - | Hypothetical protein | |
|  | 18616 | 19266 | - | Hypothetical protein | |
|  | 19268 | 19561 | - | Sp spackle periplasmic protein | |
|  | 19625 | 19873 | - | Imm Immunity to superinfection membrane protein | |
|  | 19935 | 20303 | - | Hypothetical protein | |
|  | 20374 | 20562 | - | Dmd discriminator of mRNA degradation | |
|  | 20559 | 20876 | - | Hypothetical protein | |
|  | 20965 | 22407 | - | 41 helicase | |
|  | 22417 | 22758 | - | Head formation protein | |
|  | 22751 | 23923 | - | UvsX RecA-like recombination protein | |
|  | 24023 | 24571 | - | Hypothetical protein | |
|  | 24573 | 25472 | - | Hypothetical protein | |
|  | 25472 | 26188 | - | Hypothetical protein | |
|  | 26327 | 26470 | - | Hypothetical protein | |
|  | 26467 | 27627 | - | Hypothetical protein | |
|  | 27683 | 28069 | - | Hypothetical protein | |
|  | 28069 | 29754 | - | Hypothetical protein | |
|  | 29796 | 29939 | - | Hypothetical protein | |
|  | 29939 | 30571 | - | Hypothetical protein | |
|  | 30612 | 33323 | - | DNA polymerase | |
|  | 33405 | 33773 | - | RegA, translation repressor protein | |
|  | 33776 | 34339 | - | Clamp loader small subunit | |
|  | 34341 | 35303 | - | Clamp loader small subunit | |
|  | 35379 | 36065 | - | Sliding clamp DNA polymerase accessory protein | |
|  | 36108 | 36524 | - | RNA polymerase binding protein | |
|  | 36537 | 36725 | - | Hypothetical protein | |
|  | 36780 | 38468 | - | Endonuclease subunit | |
|  | 38455 | 39315 | - | MobE mobile endonuclease | |
|  | 39302 | 39559 | - | Hypothetical protein | |
|  | 39556 | 40575 | - | Endonuclease subunit | |
|  | 40646 | 40819 | - | Hypothetical protein | |
|  | 40859 | 41065 | - | Hypothetical protein | |
|  | 41040 | 41363 | - | Hypothetical protein | |
|  | 41365 | 41580 | - | Hypothetical protein | |
|  | 41564 | 42121 | - | RNA polymerase sigma factor | |
|  | 42194 | 42463 | - | Hypothetical protein | |
|  | 42460 | 42687 | - | Hypothetical protein | |
|  | 42684 | 43016 | - | Hypothetical protein | |
|  | 43083 | 43322 | - | Hypothetical protein | |
|  | 43386 | 43586 | - | Hypothetical protein | |
|  | 43583 | 43717 | - | Hypothetical protein | |
|  | 43725 | 44018 | - | Hypothetical protein | |
|  | 44026 | 44274 | - | Hypothetical protein | |
|  | 44440 | 44763 | - | Hypothetical protein | |
|  | 44729 | 45046 | - | Hypothetical protein | |
|  | 45049 | 45264 | - | Hypothetical protein | |
|  | 45281 | 45382 | - | Hypothetical protein | |
|  | 45375 | 45845 | - | NrdG anaerobic NTP reductase, small subunit | |
|  | 45842 | 47173 | - | NrdD anaerobic NTP reductase, large subunit | |
|  | 47148 | 47918 | - | Putative GIY-YIG family homing endonuclease | |
|  | 48159 | 48704 | - | NrdD anaerobic NTP reductase, large subunit | |
|  | 48701 | 49174 | - | Recombination endonuclease VII | |
|  | 49213 | 49392 | - | Hypothetical protein | |
|  | 49402 | 49815 | - | Inhibitor of host Lon protease | |
|  | 49817 | 49987 | - | Hypothetical protein | |
|  | 50116 | 50472 | - | Hypothetical protein | |
|  | 50469 | 50732 | - | NrdC thioredoxin | |
|  | 50725 | 51138 | - | Hypothetical protein | |
|  | 51138 | 51443 | - | Hypothetical protein | |
|  | 51443 | 52393 | - | Hypothetical protein | |
|  | 52456 | 53391 | - | Hypothetical protein | |
|  | 53447 | 54442 | - | Hypothetical protein | |
|  | 54505 | 55032 | - | Hypothetical protein | |
|  | 55091 | 55480 | - | Hypothetical protein | |
|  | 55518 | 56498 | - | Hypothetical protein | |
|  | 56568 | 56786 | - | Hypothetical protein | |
|  | 56891 | 57877 | - | Hypothetical protein | |
|  | 57877 | 58350 | - | Hypothetical protein | |
|  | 58360 | 58890 | - | Hypothetical protein | |
|  | 58892 | 59071 | - | Hypothetical protein | |
|  | 59068 | 59178 | - | Hypothetical protein | |
|  | 59236 | 59442 | - | Hypothetical protein | |
|  | 59450 | 59629 | - | Hypothetical protein | |
|  | 59731 | 60033 | - | rI lysis inhibition regulator, membrane protein | |
|  | 60046 | 60258 | - | Hypothetical protein | |
|  | 60286 | 60867 | - | Tk thymidine kinase | |
|  | 60860 | 61057 | - | Hypothetical protein | |
|  | 61054 | 61245 | - | Hypothetical protein | |
|  | 61236 | 61448 | - | Hypothetical protein | |
|  | 61445 | 61912 | - | Hypothetical protein | |
|  | 61905 | 62252 | - | Vs valyl-tRNA synthetase modifier | |
|  | 62249 | 62791 | - | Hypothetical protein | |
|  | 62800 | 63258 | - | RegB site-specific RNA endonuclease | |
|  | 63321 | 63536 | - | Hypothetical protein | |
|  | 63536 | 63802 | - | Hypothetical protein | |
|  | 63792 | 64016 | - | Hypothetical protein | |
|  | 64016 | 64378 | - | Hypothetical protein | |
|  | 64385 | 64693 | - | Hypothetical protein | |
|  | 64690 | 65193 | - | Hypothetical protein | |
|  | 65403 | 65732 | - | Hypothetical protein | |
|  | 65771 | 66349 | - | Putative IpIII internal head protein | |
|  | 66413 | 66673 | - | Hypothetical protein | |
|  | 66680 | 67093 | - | DenV endonuclease V, N-glycosylase UV repair enzyme | |
|  | 67152 | 67433 | - | Hypothetical protein | |
|  | 67430 | 67918 | - | e Lysozyme murein hydrolase | |
|  | 67953 | 68411 | - | NudE nudix hydrolase | |
|  | 68408 | 68806 | - | Hypothetical protein | |
|  | 68888 | 69574 | - | Hypothetical protein | |
|  | 69576 | 69881 | - | Hypothetical protein | |
|  | 69953 | 70909 | - | Hypothetical protein | |
|  | 70934 | 71173 | - | Hypothetical protein | |
|  | 71236 | 71433 | - | Hypothetical protein | |
|  | 71443 | 71715 | - | Hypothetical protein | |
|  | 71799 | 72314 | - | Hypothetical protein | |
|  | 72319 | 72609 | - | Hypothetical protein | |
|  | 72606 | 72968 | - | Hypothetical protein | |
|  | 72970 | 73155 | - | Hypothetical protein | |
|  | 73225 | 73533 | - | Hypothetical protein | |
|  | 73594 | 73866 | - | Hypothetical protein | |
|  | 73945 | 74400 | - | Hypothetical protein | |
|  | 74400 | 74630 | - | Chaperone long and short tail fiber assembly | |
|  | 74635 | 75369 | - | Deoxynucleoside monophosphate kinase | |
|  | 75423 | 76007 | - | Tail completion and sheath stabilizer protein | |
|  | 76110 | 76931 | - | DNA end protector protein | |
|  | 76934 | 77383 | - | Head completion protein | |
|  | 77445 | 78020 | + | Baseplate wedge subunit | |
|  | 78074 | 79753 | + | Baseplate hub subunit and tail lysozyme | |
|  | 79799 | 80272 | + | Hypothetical protein | |
|  | 80275 | 80568 | + | Hypothetical protein | |
|  | 80577 | 82547 | + | Baseplate wedge subunit | |
|  | 82544 | 85642 | + | Baseplate wedge initiator | |
|  | 85635 | 86639 | + | Baseplate wedge subunit | |
|  | 86702 | 87565 | + | Baseplate wedge tail fiber connector | |
|  | 87565 | 89379 | + | Baseplate wedge subunit and tail pin | |
|  | 89379 | 90038 | + | Baseplate wedge subunit and tail pin | |
|  | 90035 | 91591 | + | Short tail fibers | |
|  | 91601 | 93043 | + | Fibritin neck whisker protein | |
|  | 93076 | 94002 | + | Neck protein | |
|  | 94004 | 94639 | + | Homing endonuclease | |
|  | 94626 | 95390 | + | Neck protein | |
|  | 95491 | 96264 | + | Tail sheath stabilizer and completion protein | |
|  | 96274 | 96768 | + | Small terminase protein | |
|  | 96752 | 98587 | + | Large terminase protein | |
|  | 98618 | 100600 | + | Tail sheath protein | |
|  | 100713 | 101204 | + | Tail tube protein | |
|  | 101289 | 102860 | + | Portal vertex protein | |
|  | 102860 | 103090 | + | Prohead core protein | |
|  | 103090 | 103515 | + | Prohead core protein | |
|  | 103515 | 104156 | + | Prohead core scaffolding protein and protease | |
|  | 104190 | 105002 | + | Prohead core protein | |
|  | 105020 | 106588 | + | Major capsid protein | |
|  | 106651 | 106917 | - | Hypothetical protein | |
|  | 107021 | 108304 | + | Capsid vertex protein | |
|  | 108334 | 109332 | - | RNA ligase 2 | |
|  | 109452 | 109703 | - | Hypothetical protein | |
|  | 109700 | 109894 | - | Hypothetical protein | |
|  | 109993 | 111123 | - | Putative large head outer capsid protein | |
|  | 111133 | 111801 | - | Minor capsid protein inhibitor of protease | |
|  | 111852 | 113366 | + | UvsW helicase | |
|  | 113392 | 113625 | + | Hypothetical protein | |
|  | 113683 | 113850 | - | Hypothetical protein | |
|  | 113883 | 114107 | - | Hypothetical protein | |
|  | 114107 | 114601 | - | Recombination, repair and ssDNA binding protein | |
|  | 114601 | 114999 | - | Baseplate wedge subunit | |
|  | 114999 | 115625 | - | Baseplate hub subunit | |
|  | 115673 | 116425 | + | Baseplate hub assembly protein | |
|  | 116422 | 117594 | + | Baseplate hub subunit | |
|  | 117605 | 118075 | + | Baseplate distal hub | |
|  | 118072 | 119844 | + | Baseplate hub subunit tail length determinator | |
|  | 119853 | 120962 | + | Baseplate subunit | |
|  | 120962 | 121924 | + | Baseplate subunit | |
|  | 121962 | 122255 | - | Hypothetical protein | |
|  | 122314 | 124401 | - | ADP-ribosyltransferase | |
|  | 124457 | 124645 | - | Hypothetical protein | |
|  | 124645 | 126138 | - | DNA ligase | |
|  | 126135 | 126404 | - | Hypothetical protein | |
|  | 126414 | 127229 | - | Hypothetical protein | |
|  | 127226 | 127684 | - | Hypothetical protein | |
|  | 127677 | 127895 | - | Hypothetical protein | |
|  | 127885 | 128082 | - | Hypothetical protein | |
|  | 128277 | 128753 | - | Hypothetical protein | |
|  | 128789 | 129154 | - | Hypothetical protein | |
|  | 129285 | 129464 | - | Hypothetical protein | |
|  | 129733 | 129981 | - | rIII lysis inhibition accessory protein, rapid lysis phenotype | |
|  | 130106 | 130438 | - | Head assembly co-chaperone for GroEL | |
|  | 130496 | 130792 | - | Hypothetical protein | |
|  | 130794 | 131375 | - | Deoxycytidylate deaminase | |
|  | 131375 | 132367 | - | Hypothetical protein | |
|  | 132488 | 132724 | - | Hypothetical protein | |
|  | 132785 | 133060 | - | Hypothetical protein | |
|  | 133068 | 133292 | - | Hypothetical protein | |
|  | 133273 | 133464 | - | Hypothetical protein | |
|  | 133464 | 134363 | - | Polynucleotide 5'-kinase and 3'-phosphatase | |
|  | 134360 | 134551 | - | Hypothetical protein | |
|  | 134551 | 134850 | - | Hypothetical protein | |
|  | 134908 | 135126 | - | Hypothetical protein | |
|  | 135123 | 135413 | - | Hypothetical protein | |
|  | 135410 | 135766 | - | Hypothetical protein | |
|  | 135754 | 136254 | - | Inhibitor of host transcription | |
|  | 136317 | 137441 | - | RNA ligase A | |
|  | 137497 | 137907 | - | Endonuclease II | |
|  | 137934 | 139109 | - | NrdB aerobic NDP reductase, small subunit | |
|  | 139163 | 141418 | - | Ribonucleoside-diphosphate reductase subunit alpha | |
|  | 141506 | 141760 | - | Hypothetical protein | |
|  | 141762 | 141995 | - | dTMP thymidylate synthase | |
|  | 142221 | 142958 | - | Homing endonuclease | |
|  | 143087 | 143638 | - | dTMP (thymidylate) synthase | |
|  | 143635 | 144222 | - | Frd dihydrofolate reductase | |
|  | 144225 | 144473 | - | Hypothetical protein | |
|  | 144535 | 144927 | - | Hypothetical protein | |
|  | 145041 | 145943 | - | Single-stranded DNA binding protein | |
|  | 146058 | 146711 | - | Hypothetical protein | |
|  | 146708 | 147046 | - | Late promoter transcription factor | |
|  | 147024 | 147293 | - | Double-stranded DNA binding protein | |
|  | 147301 | 148218 | - | RnaseH | |
|  | 148322 | 152155 | + | Long tail fiber proximal subunit | |
|  | 152164 | 153288 | + | Tail fiber hinge | |
|  | 153348 | 154004 | + | Hinge connector of long tail fiber distal connector | |
|  | 154013 | 157234 | + | Long tail fiber distal subunit | |
|  | 157271 | 158056 | + | Long tail fiber assembly catalyst | |
|  | 158102 | 158761 | + | t holin lysis mediator | |
|  | 158762 | 159034 | - | Anti-sigma 70 protein | |
|  | 159081 | 159242 | - | Hypothetical protein | |
|  | 159232 | 159531 | - | Hypothetical protein | |
|  | 159606 | 159833 | - | Hypothetical protein | |
|  | 159817 | 160098 | - | Hypothetical protein | |
|  | 160098 | 160559 | - | Hypothetical protein | |
|  | 160556 | 160885 | - | Hypothetical protein | |
|  | 160896 | 161528 | - | MotA activator of middle period transcription | |
|  | 161714 | 163039 | - | DNA topoisomerase II medium subunit | |
|  | 163044 | 163184 | - | Hypothetical protein | |
|  | 163181 | 163330 | - | Ac acridine resistance protein | |
|  | 163383 | 163829 | - | Nucleoid disruption protein | |
|  | 163906 | 164106 | - | Hypothetical protein | |
|  | 164357 | 164620 | - | Hypothetical protein | |
|  | 164689 | 165159 | - | DNA endonuclease IV | |
|  | 165182 | 165469 | - | Hypothetical protein | |
|  | 165513 | 166448 | - | rIIB protector from prophage-induced early lysis | |
